# Normalizing Tumor Vasculature to Reduce Hypoxia, Enhance Perfusion, and Optimize Therapy Uptake

**DOI:** 10.3390/cancers13174444

**Published:** 2021-09-03

**Authors:** Kathy Matuszewska, Madison Pereira, Duncan Petrik, Jack Lawler, Jim Petrik

**Affiliations:** 1 Department of Biomedical Sciences, University of Guelph, Guelph, ON N1G 2W1, Canada; kmatusze@uoguelph.ca (K.M.); mperei02@uoguelph.ca (M.P.); petrikd@uoguelph.ca (D.P.); 2 Beth Israel Deaconess Medical Center, Harvard Medical School, Boston, MA 02215, USA; jlawler@bidmc.harvard.edu

**Keywords:** hypoxia, angiogenesis, vascular normalization, drug delivery, therapy resistance

## Abstract

**Simple Summary:**

In order for solid tumors to grow, they need to develop new blood vessels in order to support their increasing metabolic requirements. To facilitate the novel vessel formation, the tumor initiates an aggressive pro-angiogenic program. As a result of the aggressive angiogenesis, blood vessels form very rapidly and are often malformed and dysfunctional. There is a reduction in perfusion to the tumor, and often the tumors exhibit significant areas of tumor hypoxia. This review paper discusses the pro-tumorigenic environment induced by tumor hypoxia and how this can be targeted through normalization of the tumor vasculature. Here, we review tumor angiogenesis, the development of a hypoxic phenotype, and how this contributes to sustained tumorigenesis and resistance to therapy. We further discuss the potential of vascular normalization to reduce tumor hypoxia and facilitate uptake and efficacy of a variety of therapies.

**Abstract:**

A basic requirement of tumorigenesis is the development of a vascular network to support the metabolic requirements of tumor growth and metastasis. Tumor vascular formation is regulated by a balance between promoters and inhibitors of angiogenesis. Typically, the pro-angiogenic environment created by the tumor is extremely aggressive, resulting in the rapid vessel formation with abnormal, dysfunctional morphology. The altered morphology and function of tumor blood and lymphatic vessels has numerous implications including poor perfusion, tissue hypoxia, and reduced therapy uptake. Targeting tumor angiogenesis as a therapeutic approach has been pursued in a host of different cancers. Although some preclinical success was seen, there has been a general lack of clinical success with traditional anti-angiogenic therapeutics as single agents. Typically, following anti-angiogenic therapy, there is remodeling of the tumor microenvironment and widespread tumor hypoxia, which is associated with development of therapy resistance. A more comprehensive understanding of the biology of tumor angiogenesis and insights into new clinical approaches, including combinations with immunotherapy, are needed to advance vascular targeting as a therapeutic area.

## 1. Introduction

### 1.1. Sprouting Angiogenesis in Normal Physiology

Angiogenesis is the complex and highly regulated formation and maturation of vasculature from pre-existing vessels throughout the body. Typically, the process is kept quiescent through a balance of growth factors and inhibitors. Normal human processes that necessitate angiogenesis in the adult include placentation in the pregnant uterus, formation of the endometrium in the menstrual cycle, growth of the mammary gland in preparation for lactation, and supply of granulation tissue for wound healing [1,2,3]. In any of these situations, angiogenesis consists of a series of events including removal of structural pericytes in the area of the developing sprout, degradation of the capillary basement membrane, migration and proliferation of the endothelial cells comprising the new sprout, nascent tube formation, and vascular stabilization [4].

**Figure 1:**
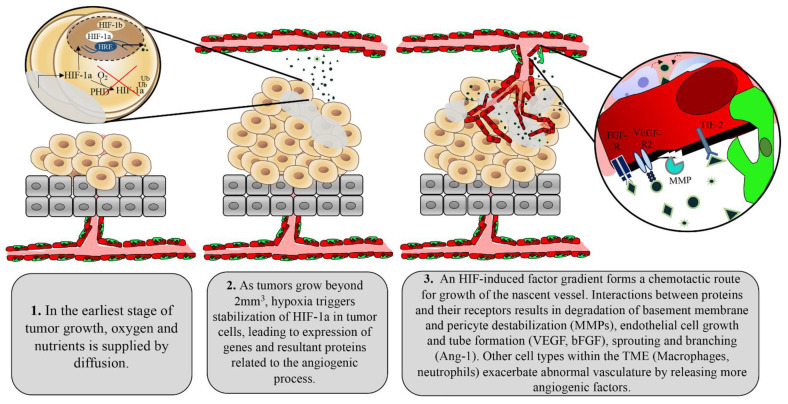
Hypoxia induced by the growing tumor mass triggers an “angiogenic switch” within the tumor microenvironment, resulting in a crude version of angiogenesis.

The presence of angiogenic stimuli such as hypoxia, mechanical stress, or inflammation leads to the release of growth factors, as summarized in Figure 1. These signaling events ultimately lead to the activation of cellular effectors, which aim to form the nascent vessel [4]. Upon effector stimulation, smooth muscle cells called pericytes located at intervals along the capillary wall are first removed from the sprouting area of a mother vessel. VEGF stimulation triggers intricate calcium oscillations within endothelial cells allowing for the selection of an endothelial cell distinguished by specialized filopodia, called a tip cell [5]. The tip cell guides the developing sprout through chemotaxis, following angiogenic stimuli secreted by the target tissue requiring increased perfusion [5]. As tip cells are highly influenced by even minute fluctuations in growth factor signaling, a loss of growth factor balance in this system may lead to disorganized vasculature. The tip cell releases matrix metalloproteases (MMP), which degrade basement membrane components in its path [6]. A second group of specialized endothelial cells, called stalk cells, are highly proliferative and interact with tip cells through delta-notch signaling to elongate the nascent sprout [7]. At a point of anastomoses between the tip cell of another nascent vessel or stabilized vessel, junctional adhesion proteins are deposited at the contact site of the two tip cells. A lumen is formed through cell membrane invagination or cord hollowing, forming a functional vascular network [8]. Circulating endothelial progenitor cells also contribute to the nascent vessels, which are haphazardly branched and in need of organization. Local differences in blood flow and pressure lead to the elimination of poorly perfused branches (pruning) or recycling of their component endothelial cells to areas of significant flow [9,10]. Conversely, highly perfused sprouts are stabilized through deposition of basement membrane, reduced endothelial cell activity, tightening of cell junctions, and recruitment of pericytes [10].

### 1.2. Tumor Control of Angiogenesis

In many ways, tumors can be considered functional organs as opposed to a group of aberrant cells. The tumor stroma includes mesenchymal-derived cells, inflammatory cells, and vascular cells, albeit in an irregular fashion that has been modified by the tumor to tailor to its survival needs [11,12]. Tumors are therefore capable of inducing angiogenesis by co-opting the same pro-angiogenic program. Small tumors devoid of vasculature are often observed in solid tumor types—their oxygen and nutrient demands being supplied by passive diffusion from nearby vessels [13]. However, as tumors grow beyond 2 mm^2^, the tumor core becomes increasingly hypoxic and the process of angiogenesis begins to fuel oxygen and nutrient demands [14]. This moment has been termed “the angiogenic switch” in which tumor cells respond to low oxygen perfusion by releasing many of the angiogenic factors represented in Figure 1 [15]. Cellular responses to low oxygen are primarily regulated by DNA-binding transcription factors known as hypoxia inducible factors (HIF). HIFs are heterodimeric proteins that consist of a constitutively expressed HIF-1ß subunit and an oxygen-regulating subunit (HIF-1α or HIF-2α) [16,17]. These alpha subunits are composed of an amino terminal basic Helix-Loop-Helix (bHLH) necessary for DNA binding to hypoxia response elements (HRE), transactivation domains (N-TAD and C-TAD) that are vital for activation of HIF target genes, PAS-A and PAS-B domains for protein-protein dimerization, and an oxygen-dependent degradation domain (ODDD). Redundancy in HIF-1α stabilization is evident as a secondary lysine residue within the ODDD can be acetylated by an acetyl transferase enzyme called arrest-defective-1 (ARD-1) to favour degradation of HIF-1α [18]. The expression of ARD-1 is decreased in hypoxia, resulting in stabilized HIF-1α under this condition [18].

Under normoxic conditions, the prolyl hydroxylase domain (PHD) uses oxygen as a rate-limiting substrate and iron as a cofactor to hydroxylate two proline residues within the ODDD [18]. Hydroxylated HIF-1α becomes associated with Von Hippel Lindau factor (pVHL) and elongins B and C, cullin-2 (cul-2) and rbx1 co-factors, forming a complex with E3 ubiquitin ligase activity (HIF-1α-VBC complex) [19]. However, under hypoxic conditions, HIF-1α is stabilized through limited PHD activity. This allows generation and accumulation of non-hydroxylated HIF-1α. Further, HIF-1α stability is controlled by ubiquitin ligases that are PHD enzymes themselves, as well as pVHL-interacting deubiquitinating enzyme (VDU2), which acts to destabilize ubiquitin ligases on HIF-1α [18]. Given the significantly short half-life of HIF-1α (<1 min in a perfused lung), it is constantly being degraded at physiological oxygen levels in normal cells and is subject to tight regulation should oxygen levels decline [20]. In contrast, the median oxygenation of an untreated tumor falls between approximately 0.3% and 4.2%, with most untreated tumors exhibiting median oxygen levels <2% [21]. This level of hypoxia triggers the release and stabilization of HIF-1α while also inducing oncogenic mechanisms that further derail the HIF pathway and make tumors less dependent on oxygen [21]. Tumor-induced mutations in the binding pocket of pVHL have been shown to disrupt HIF-1α interactions and thereby disassemble the E3 ubiquitin ligase (VEC) complex [22]. More directly, in lung cancer, *TP53* mutants have been shown to exert a gain of function on HIF-1, leading to heightened expression of hypoxia-response genes [23]. HIF-1α is capable of binding directly to the tumor suppressor, favouring mouse double minute 2 homolog (Mdm2) ubiquitination and proteosomal degradation of HIF-1α, which is not possible in TP53 mutants or knockouts [24]. Several studies have shown that HIF expression is abrogated upon phosphoinositide 3-kinase (PI3K) pathway inhibition regardless of oxygen levels [25,26]. Similarly, HIF-1 is upregulated by AKT in human gastric cancer, breast cancer, and non-small cell lung cancer [27,28].

### 1.3. Factors Contributing to Tumor Vascular Dysfunction

Tumors initiate the angiogenic process through activation of multiple factors including the most prominent angiogenic ligand, vascular endothelial growth factors (VEGF), and its receptors including VEGFR2 [29,30,31]. The VEGF family of proteins includes VEGF-A, VEGF-B, VEGF-C, VEGF-D, VEGF-E, and placenta growth factor (PIGF) [31]. VEGF-C and VEGF-D are studied as regulators of lymphangiogenesis, while VEGF-A is commonly referred to simply as VEGF due to its dominant role in angiogenesis. VEGF undergoes alternative splicing, leading to several isoforms that differ based on heparin binding affinity, localization to the extracellular matrix, or diffusive potential. The VEGF gene is transcriptionally regulated in response to HIF, and its levels must be tightly controlled to prevent aberrant angiogenesis [31]. Due to their control over HIF, tumor cells release exaggerated levels of VEGF to the extracellular space in response to hypoxia [32]. High concentrations of VEGF surrounding endothelial cells select for excess tip cells, which then contribute to irregular branching and tortuous vascular networks. The basement membrane of tumor vessels, which serves as a physical barrier for cancer cell metastasis to surrounding tissues, is often absent or thin due to chemical degradation by tumor-derived proteases [33,34]. The monolayer of endothelial cells is often disorganized and cells are plagued with abnormal gene expression profiles, karyotypic abnormalities, and chromosomal instability [35,36,37]. Compared with normal endothelial cells, tumor endothelial cells contain four times the amount of total RNA, indicating enhanced gene expression. Indeed, tumor endothelial cells have enhanced expression of VEGFR-1 and -2 and are therefore more responsive to VEGF stimulation [38]. Recently, tumor endothelial cells have been shown to have enhanced expression of markers of angiogenesis and stemness such as CD61, CD105, Sca-1, CD34, CD90, and ALDH [39,40]. These expression profiles contribute to the escalated angiogenic potential of tumor endothelial cells compared with normal endothelial cells, which facilitates the aberrant vascular arrangement seen in tumors [41]. Extracellular factors such as VEGF, PMA, TGF-ß, and cytochalasin B, which are overexpressed in the tumor microenvironment, have been shown to impact fenestration formation in endothelial cells [42,43]. Given that these plasma membrane microdomains are vital for the exchange of solutes and water at the interface of tissue and vasculature, tumor endothelial cells are often more porous compared with normal counterparts [43]. Abnormal VEGF signaling in tumor endothelial cells also leads to downregulation of connexin expression, causing gap junction dysfunction, increasing vascular fenestrations, and increasing vascular permeability [44,45,46]. In fact, VEGF was initially identified based on its ability to increase vascular permeability and extravasation of plasma proteins, such as fibrinogen [47].

Pericytes are specialized smooth muscle cells that are recruited to mature and stabilized vessels through release of PDGF-ß by ECs [48]. Mice deficient in PDGF-ß signaling lack pericytes and succumb to micro hemorrhaging, demonstrating the importance of these cells for proper vascular function [48,49]. Signals secreted by pericytes maintain EC survival by leading to enhanced expression of BCL-w antiapoptotic protein [49]. Pericytes therefore also shelter normal vessels from anti-angiogenic therapies, allowing for tumor-targeted action of these agents. Hypoxia and downstream angiogenic factors released by tumor cells disengage pericytes from endothelial cells as the initial step to the formation of the nascent vascular sprout. Therefore, tumor-associated vessels are largely devoid of pericytes or demonstrate weak connections between pericytes and endothelial cells, contributing to an immature vascular phenotype and facilitating continued angiogenesis [50].

### 1.4. Abnormal Vasculature Results in Limited Treatment Delivery

The abnormalities of the tumor vasculature result in poor tissue perfusion, which poses a physical barrier to therapy delivery to tumors. Of the number of delivery and uptake impediments, elevated interstitial pressure (IFP) is considered to be the most significant barrier to therapy access to the tumor [51,52,53]. The etiology of IFP elevation is multifactorial and involves high vascular permeability and mechanical compression of lymphatic blood vessels [54,55]. Disrupted vascular morphology with reduced pericyte coverage is associated with a loss of endothelial cell junction integrity and an activated endothelium, resulting in vessels that are leaky and extravasate fluid into the tumor environment, thereby increasing pressure within the tumor [56,57]. Combined with solid stress, in which accumulation of cancer cells, stromal cells, cancer-associated fibroblasts (CAFs), and their associated extracellular matrix create high mechanical pressure within the tumor, IFP leads to a significant elevation in intratumoral pressure [58].

This high IFP causes a stasis in flow throughout the tumor, which results in tumor hypoxia and acidosis [59]. Elevated hypoxia as a consequence of high IFP is associated with poor outcome in cancer patients and is considered an early response marker for cancer therapeutics such as chemotherapy and radiation [60,61]. As another consequence of reduced perfusion and flow within the tumor, there is impediment of drug uptake and delivery within the tumor tissue [62]. With the elevated IFP, there is an attenuated transvascular osmotic pressure difference, resulting in impaired delivery of drugs throughout the tumor [63]. Even in tumors in which there is vascular heterogeneity, drugs will become concentrated in regions that have sufficient blood supply but will have limited migration to areas in which IFP is higher and vessel density is decreased [64]. Although IFP is often discussed in relation to the primary tumor, it is important to note that larger metastatic tumors also demonstrate elevated IFP and decreased drug uptake, potentially contributing to the development of drug-resistant metastatic disease.

**Figure 2:**
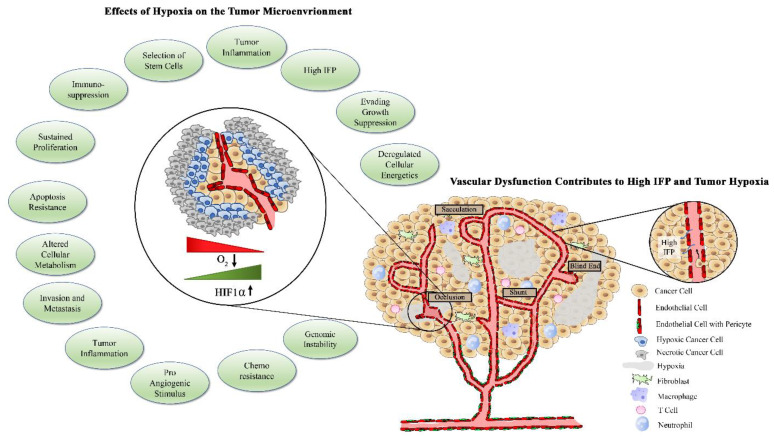
Tumor hypoxia activates several tumorigenic processes. Tumor vasculature has altered morphology, with reduced pericyte coverage. The immature tumor vessels are characterized by blind end shunts, torturous pathway, sacculations, decreased luminal size, and increased fenestrations. Excessively fenestrated vessels allow for fluid extravasation and increased interstitial fluid pressure (IFP) and facilitate intravasation and migration of metastatic tumor cells. Elevated IFP and disrupted tissue perfusion contribute to areas of acute and chronic hypoxia, which can activate numerous pro-tumorigenic processes.

While intra-tumoral treatment delivery decreases off-target toxicities, it fails to account for metastatic disease and has not led to significant survival benefit compared to systemic administration [65]. Clinical use of intra-tumoral drugs is also impractical for some tumor subtypes such as ovarian and pancreatic cancers, which are inaccessible through transdermal injection. In order to prove effective, systemic agents must not only navigate from the injection site to the tumor vasculature but must also gain access and disperse throughout a tumor, which is often plagued with impediments to this process, posing a therapeutic challenge [66]. The properties of the tumor microenvironment that pose issues for treatment delivery are depicted in Figure 2.

### 1.5. Hypoxia and Tumor Metabolism

As tumors grow beyond their vascular supply, skewed supply and demand of oxygen and heterogenic blood supply contribute to the development of an O_2_ gradient within the tumor. In this setting, rapidly proliferating peripheral cells consume available oxygen supplied by vessels, thus limiting oxygen diffusion to the core. This heterogenous distribution of oxygen and subsequent hypoxic regions select for an aggressive phenotype and fuel metastasis as well as treatment resistance, which has been extensively reviewed [67,68]. Hypoxia can alter metabolic pathways within the tumor cells including the impairment of oxygen-dependent processes of mitochondrial oxidative phosphorylation, hindering ATP production. As a result, the tumor cells rewire their metabolism from oxidative phosphorylation to aerobic glycolysis through a process known as the Warburg Effect [69]. Here, a significant increase in glucose consumption results in excess production of lactate [67]. Tumor cells are able to produce ATP at a much more rapid rate through glycolysis and become addicted to glucose consumption to aid in their ability to proliferate at a higher rate compared with normal cells.

With the tumor cells consuming copious amounts of glucose in order to survive and proliferate, an accumulation of lactate occurs. While hypoxic tumor cells favour glucose consumption, normoxic tumor cells, which are located closest to the vasculature, have the option to consume glucose or lactate to fuel their metabolics [70]. Interestingly, these normoxic cells choose to use lactate over glucose, and the accumulated lactate waste produced by hypoxic tumor cells is recycled and reused by normoxic tumor cells, fueling metabolic symbiosis between the tumor cells and the tumor microenvironment [71].

When glucose levels are low, excess lactate signals oxidative-tumor cells to use glutaminolysis, another metabolic avenue that tumor cells rely heavily on to fuel their energy source, as glutamine is imperative to assist rapidly proliferating tumor cells [72]. Cancer cells also require a strong demand for NADPH and many other biosynthetic precursors making glutaminolysis a perfect avenue to maintain the tricarboxylic acid (TCA) cycle and replenish these depleted energy ATP levels. In addition to ATP production, lactate is also produced further contributing to the metabolic symbiosis in the tumor microenvironment [73].

Extracellular lactate accumulation as a result of tumor aerobic glycolysis and glutaminolysis leads to the development of an acidic tumor microenvironment, thereby influencing the potentiality of more aggressive and invasive tumor cells [74]. Extracellular acidic environments have been shown to manipulate gene expression, induce G1 cell cycle arrest, and increase necrotic cell death [75]. This acidity can result in a more aggressive phenotype of tumor cells, primarily through affecting their invasive capacity and metastatic potential [76]. Similar to hypoxic conditions, by maintaining a low pH, tumor cells are able to evade surveillance and destruction by immune cells [77]. Additionally, a low pH in the tumor microenvironment is a known contributor to more aggressive tumor phenotypes and chemoresistance [78].

### 1.6. Hypoxia and Drug Resistance

A number of cancer treatment strategies rely on the presence of oxygen in order to exert their anti-tumor effect. The basis of radiotherapy is generation of reactive oxygen species, which then damages tumor cell DNA resulting in cell death. This reaction in turn becomes permanent when oxygen reacts with the free electron of the free radical. In a pioneering study, Gray and colleagues demonstrated that the presence of oxygen conferred radiation sensitivity in tumors. In fact, killing hypoxic tumor cells requires a three-fold higher dose of radiation compared with killing normoxic tumor cells [79]. This is problematic for treatment success given that the dose of radiation cannot be safely increased to compensate for this difference in light of limited radiation tolerance of normal tissues. Indeed, normoxic tumors have a higher chance of radiotherapy success. Likewise, photodynamic therapy (PDT) relies on oxygen to induce photo-oxidation, and PDT resistance is common in hypoxic tumors [80,81].

The role of hypoxia in chemoresistance is well-documented and involves increased HIF production in response, which enhances the expression of membrane efflux pumps. The most common efflux pumps linked to multidrug resistance are the ATP Binding Cassette (ABC) family of transporters, which reduce intracellular accumulation of chemotherapy to sub-therapeutic levels [82]. Although constantly expressed, HIFs are degraded when there is normal oxygen tension, but are stabilized in the presence of hypoxia [18]. In colon cancer cells subjected to hypoxia, HIF-1 activation occurred, which resulted in overexpression of multidrug resistance 1 (MDR1; P-glycoprotein) [16,83]. MDR1 is an ATP-dependent efflux pump that can effectively transport chemotherapy drugs out of the cell and is one of the major mechanisms involved in chemotherapy failure [84,85]. HIF-1 upregulation of MDR1 is also associated with chemoresistance, enriched stem cell population, and aggressive phenotype in triple negative breast cancers [86]. In colon cancer cells, blocking HIF-1α has been shown to reverse multi-drug resistance via downregulation of P-glycoprotein [87]. In the presence of hypoxia, HIF-1α increases the activity of Snail and Twist, transcription factors that promote epithelial-to-mesenchymal transition (EMT) and are associated with resistance to chemotherapy [88]. Several groups have found that hypoxic tumor cells are less proliferative than their normoxic counterparts [89]. This becomes problematic given that chemotherapy targets highly proliferative cells, thereby selecting for the survival of the more aggressive hypoxic cells. Further, Saggar et al. found that chemotherapy repopulates hypoxic cells that contribute to treatment failure, possibly due to enhanced nutrient availability following clearance of rapidly proliferating cells [90]. The link between normoxia and chemotherapeutic success likely explains the benefits of hyperbaric oxygen therapy (HBOT) in improving their effectiveness [91,92,93]. The excess oxygen molecules provided by HBOT enhances chemotherapy-induced oxidative stress, thereby lowering the therapeutic dose and mitigating side effects [91,92,93].

### 1.7. Hypoxia and the Immune Environment

Immunotherapy has become the fourth pillar of cancer therapy, joining surgery, radiation, and chemotherapy. Hypoxia has been identified as a barrier to the success of immunotherapy due to its association with tumor escape from immune detection [93]. Through stabilization of HIF-1, hypoxia upregulates chemokines such as CCL28, which enhances tumor influx and function of myeloid-derived suppressor cells [94]. HIF-1α also increases the expression of forkhead box P3 (FoxP3), which is indispensable for the development of Tregs [95]. Hypoxia also promotes immune evasion by upregulating expression of checkpoint molecules such as the programmed death ligand 1 (PD-L1) [96] and involves binding of HIF-1 to a hypoxia response element in the PD-L1 proximal promoter [97]. Hypoxia is also reported to inhibit the antitumor immune response. HIF-1α stabilization prevents TCR-mediated Ca^2+^ signaling and prevents CD8+ T cell activation [98]. In mice, low oxygen availability led to the reduction of aggressive cellular activity and correlated with decreased pro-inflammatory cytokine production [99]. Likewise, areas of tumor hypoxia are associated with reduced T lymphocyte proliferation and enhanced apoptosis and are often relatively devoid of these cells [100]. In addition to the effects on T lymphocytes, hypoxia also affects the function of natural killer (NK) cells. There is substantial evidence that hypoxia suppresses the cytotoxic effect of NK cells in tumors [101]. Upregulation of HIF-1α within the tumor can lead to decreased expression of the natural killer group 2 member D (NKG2D) receptor on NK and T cells, leading to immune evasion and impaired tumor cell killing [101,102]. Hypoxia is also known to enhance the uptake of regulatory T-cells (Tregs), which lead to the activation of transforming growth factor-beta (TGF-ß), further suppressing NK cell function [103]. TGF-ß is also a key player in the recruitment of cancer-associated fibroblasts (CAFs) in solid tumors [104]. CAFs are responsible for production of cytokines and generation of fibrous material, which contributes to mechanical barriers to immune cell infiltration and function [105]. Dendritic cells, the main antigen-presenting cells, are critical in activating naïve T cells and generating a specific immune response [103]. Sustained HIF-1α expression led to DC expression of immunosuppressive mediators such as iNOS and IL-10, which hindered CD8 T cell function [105]. Through gene knockout studies in mice, Weigert et al. demonstrated that HIF-1α hinders the generation of dendritic cells in the bone marrow [106].

### 1.8. Therapeutic Use of Vascular-Targeting Agents

The poor prognosis of treatment strategies in hypoxic tumors has prompted studies into identifying oxygenation status of tumors as a way to predict therapy efficacy [107]. Several strategies have been employed to use hypoxia as an advantage to therapy. For instance, hypoxia-activated prodrugs (HAPs) are enzymatically reduced in low oxygen levels to generate cytotoxic species [108]. Other strategies focus on reversing tumor hypoxia, such as enhancing the oxygen-carrying capacity of plasma through hyperbaric oxygen therapy [109]. Moreover, molecules that improve the rate of diffusion of oxygen from red blood cells to the vascular wall [110] and engineered oxygen transport molecules [111] are yielding promising results as combination therapies, preclinically [112]. Further strategies focus on targeting the source of tumor hypoxia: tumor vasculature. In 1993, Kim et al. formed murine tumor models of rhabdomyosarcoma, glioblastoma, and leiomyosarcoma and found that mice treated with VEGF monoclonal antibodies suppressed tumor growth [113]. Given that the antibodies had no effect on these cells in vitro, this pioneering study demonstrated that blocking the actions of an angiogenic mediator has direct effects on tumor growth by manipulating tumor vasculature. Early studies into anti-angiogenic agents were designed to induce destruction of the tumor vessels in hopes of starving the tumor. Although vascular disruption yields acute anti-tumor effects, this extensively reviewed strategy does not translate to long-lasting tumor suppression [113,114]. Several anti-angiogenic therapies have been approved clinically, although their benefit to overall survival has been modest likely due to the aggressiveness of cancer cells as they adapt to lower oxygen levels in their environment [115]. Godet et al. (2019) [92] demonstrated that lung cancer cells exposed to hypoxia in the primary tumor environment are six times more likely to become viable circulating cells compared with those in normoxic tumor areas [92,116]. Hypoxic cells develop a gene signature that includes changes in p53 and e-cadherin—ensuring their resistance to oxidative stress and fueling metastasis [117]. Even following re-oxygenation, the cells exhibited “hypoxic memory” and maintained this aggressive phenotype [117]. High-dose anti-angiogenic therapies also have undesirable effects such as further reduction of oxygen levels and decreased tumor delivery of chemotherapy due to further vessel destruction and greater impairment of tumor perfusion. These agents have instead been evaluated in low doses as adjuvants to chemotherapy, for which they have garnered success clinically, as demonstrated in Table 1. This efficacy is largely attributed to a phenomenon known as vascular normalization, proposed by Jain et al. in 2001 [118,119]. The process of vascular normalization involves improving overall morphology of vasculature by specifically destroying immature vessels while maintaining intact tumor vasculature that resembles normal vessels throughout the body. The reduced demand for blood supply brought about by reliable circulation reduces HIF-1, thereby re-establishing the balance between pro- and anti-angiogenic factors. Immature vessels—those that appear tortuous with expanded lumens, display decreased pericyte coverage, and require VEGF for survival—are highly dependent on circulating angiogenic factors [120]. Their dependence makes them susceptible to anti-angiogenic molecules and are therefore pruned in the process of vascular normalization. Similarly, vasculature with low pericyte investment is also more susceptive to anti-angiogenic therapy. This is likely as a result of paracrine signaling between endothelial cells and pericytes, which maintains stability of mature vessels [121].

## 2. Considerations for Success of Vascular Normalizing Agents

The observation of improved perfusion was initially counter-intuitive in that researchers expected that anti-angiogenic therapy would decrease tumor profusion. Evidence now suggests that the clinical success of vascular normalization appears to be a function of dose, duration of treatment, and tumor subtype or vascularization status. In early clinical trials with the anti-VEGF antibody, bevacizumab, those patients whose tumors demonstrated significant improvement in perfusion following anti-angiogenic therapy showed the greatest progress clinically [114]. In fact, higher doses of, or extended treatment with, VEGF pathway antagonists can return to vascular destruction and a subsequent decrease in perfusion, leading to the concept of a “normalization window” where optimal benefit is achieved [118]. The presence of abnormal vessels is a key indicator for response to anti-angiogenic therapy because this therapy can induce hypoxia of poorly vascularized tumors. As such, high microvascular density at the beginning of treatment correlates with response to bevacizumab treatment [147]. Whereas increased overall survival was not detected in the total population of 980 ovarian cancer patients after bevacizumab treatment, increased overall survival was increased in the subpopulation of patients with increased vessel density and higher amounts of VEGF prior to treatment. A correlation between baseline microvessel density and response to anti-angiogenic therapy is also seen in breast cancer, non-small-cell lung cancer, and colorectal cancer [121,148,149]. By contrast, no correlation between baseline vascular density and response to anti-VEGF therapy was observed in renal cancer [150]. These data indicate that the efficacy of vascular normalization varies between tumor types and between patients. In addition, as discussed below, vascular normalization differs between therapeutics. Thus, there is not a “one-size-fits-all” approach to normalizing the tumor vasculature.

In addition to pruning tumor vessels, the fortification of vessels by pericytes is a key component of vascular normalization. Thus, the relative number of vessels with pericyte coverage can be used to quantify vessel normalization and response to therapy. The recruitment of pericytes can help to limit excess pruning induced by anti-VEGF treatment. Therapeutics that favour fortification over pruning may have significant benefit in that their normalization window may be wider. Thrombospondin-1 (TSP-1)-based reagents may be well-suited to increase fortification in that TSP-1 has been shown to promote smooth muscle cell proliferation and migration while inducing apoptosis of endothelial cells [151,152]. A significant portion of the anti-angiogenic activity of TSP-1 resides within the type 1 repeats of TSP-1, designated 3TSR. As described below, 3TSR has potent normalizing effects of the tumor vasculature, which in turn promotes delivery of therapeutics and immune cells to tumors. Systemic upregulation of TSP-1 reportedly mediates the anti-angiogenic effect of metronomic dosing of chemotherapy [153].

Angiopoietin-1 and its receptor Tie-2, which is present on endothelial cells and pericytes, promotes vessel maturation and fortification, and thus, resistance to anti-angiogenic therapy. Tie-2 is inactivated by angiopoietin-2 and vascular endothelial protein tyrosine phosphatase (VE-PTP) [154]. Goel et al. (2013) have shown that inhibiting the activity of VE-PTP fortifies vessels and promotes the delivery of chemotherapeutics in mammary tumors. Similarly, blocking angiopoietin-2 can promote vascular normalization and prolong survival induced by anti-VEGF therapy in glioblastoma [155].

Upregulation of Tie-2 signaling or TSP-1 represent two examples of a wide range of reagents that can regulate vascular normalization through modulating metabolism, signal transduction, and extracellular matrix degradation [121]. Similarly, recently type 1 T helper cells have been shown to participate in vascular normalization through immune reprogramming [156]. A co-dependence of the immune and vascular systems was evident from the fact that vascular normalization was decreased by depletion or inactivation of CD4+ lymphocytes. Furthermore, adoptive transfer of T_H_1 cells to immunodeficient tumor-bearing mice reduced hypoxia in immunodeficient mice. Taken together, the data indicate that vascular normalization through pruning and fortification can be achieved in multiple ways. There must be excessive vascularization prior to treatment so that the anti-angiogenic therapy does not increase hypoxia and the anti-angiogenic therapy must promote fortification in order for there to be a therapeutic benefit. Identifying optimal strategies for optimizing vascular normalization is an important area for future research.

## 3. Vascular Normalizing Agents as Adjuvants to Traditional Cancer Therapeutics

Normalized tumor vessels have also been shown to re-program many other aspects of the tumor microenvironment known to limit delivery of cancer therapies discussed earlier in this review, giving rise to the term ‘microenvironment normalization’ [99]. Anti-angiogenic drugs have opened new avenues for combination therapy. In a humanized murine model of colorectal adenocarcinoma, combination therapy with anti-PDGFR and anti-VEGFR tyrosine kinase inhibitors decreased IFP in tumors, allowing for enhanced delivery of taxol therapy [157]. Improved delivery of chemotherapy through vascular normalization in solid tumors has been extensively reviewed [100]. We and others have extended the utility of vascular normalization to enhancing the delivery and functionality of agents beyond traditional chemotherapy. The vascular shutdown typical of oncolytic viruses (OV) was prevented using thrombospondin type-1 repeats in a mouse model of advanced stage ovarian cancer [158]. This led to enhanced intratumoral trafficking of immune cell subsets, thereby improving immunotherapeutic success [99,158,159]. In addition to enhancing vascular perfusion and providing a conduit for immune cells, the enhanced oxygenation of tumors as a result of low-dose anti-angiogenic therapy has improved immune cell function and reprogramed immune cell subsets with greater anti-tumor capabilities [156]. Vascular normalizing therapies continue to be recognized for their oxygen-modulating function in sensitizing tumors to traditional therapies, which have often been met with resistance [160].

## 4. Conclusions

Dysregulated tumor vasculature creates tumor hypoxia, which encourages aggressive tumor cell adaptations, impedes immune surveillance, fuels metastasis, and promotes resistance to current standard of care treatments. Pre-clinical studies of novel therapeutic strategies often fail to account for the vascular density and oxygenation status of the tumor subtype, resulting in a lack of clinical efficacy in patients. Anti-angiogenic therapies have the potential to normalize the tumor microenvironment, allowing for significantly better anti-tumor results when used in combination with other therapeutics. Future studies should focus on optimal timing and dosing of these agents in candidate solid tumors to prevent over-normalizing or pruning back tumor vasculature when the intent is to improve perfusion for enhanced systemic therapy.

## Figures and Tables

**Table 1:** Clinical success of FDA-approved anti-angiogenic drugs alone and in combination, as measured by progression free survival (PFS) and overall survival (OS).

Anti-Angiogenic Drug	FDA Approval	Mechanism	Indication	Combination Agent	Anti-angiogenic Drug + Combination Agent vs. Combination Agent Alone (*)		Ref.
					PFS (mts)	OS (mts)	
Bevacizumab (Avastin^®^)	2004	Humanized monoclonal antibody that binds to and inhibits the activity of VEGF-A	Cervical	Paclitaxel + Cisplatin	**9.63** (* 6.67)	**17.51** (* 12.68)	[122]
				Paclitaxel + Topotecan	**7.36** (* 5.29)	**16.20** (* 12.68)	[122]
			Colorectal (metastatic)	5-Fluorouracil	**8.8** (* 5.6)	**17.9** (* 14.6)	[123]
			NSCLC	Carboplatin + Paclitaxel	**6.2** (* 4.5)	**12.3** (* 10.3)	[124]
			Ovarian, Fallopian, primary peritoneal	Carboplatin + Paclitaxel	**18.1** (* 14.5)	**36.6** (* 28.8)	[125]
			Renal Cell	Interferon alfa	**10.2** (* 5.4)	**23.3** (* 21.3)	[126,127]
Axitinib (Inlyta^®^)	2012	Tyrosine Kinase Inhibitor (VEGFR-1, VEGFR-2, VEGFR-3)	Renal Cell	Pembrolizu mab	**15.1** (* 11.1)	-	[128]
Cabozantinib (Cometriq ^®^)	2012	Tyrosine Kinase Inhibitor (VEGF, MET, AXL)	Hepatocellular	Placebo	**5.2** (* 1.9)	**10.2** (* 8.0)	[129]
			Medullary Thyroid	Placebo	**11.2** (* 4.0)	-	[130]
Everolimus (Afinitor^®^, Zortress ^®^)	2009	mTOR inhibitor	Breast	Exemestane	**10.6** (* 4.1)	-	[131]
			Advanced Kidney	Lenvatinib	**14.6** (* 7.4)	-	[132]
Pazopanib (votrient^®^)	2009	Tyrosine Kinase Inhibitor (VEGFR-1,-2, -3, PDGFR-a, -b, c-KIT, FGFR-1, -3)	Renal Cell	placebo	**9.2** (* 4.2)	**22.9** (* 20.5)	[133]
			Soft Tissue Sarcoma	placebo	**4.6** (* 1.6)	**12.5** (* 10.7)	[134,135]
Regorafenib (Stivarga^®^)	2012	Tyrosine Kinase Inhibitor (VEGFR-1, -2, -3, TIE-2, PDGRF, FGFR, KIT, RET, RAF-1, BRAF)	Colorectal Cancer	Placebo	-	**6.4** (5.0)	[135]
			Gastrointestinal	Placebo	**4.8** (* 0.9)	-	[136]
			Hepatocellular	Placebo	-	**10.6** (* 7.8)	[137]
Sorafenib (Nexavar^®^)	2005	Tyrosine Kinase Inhibitor (Raf, PDGF, VEGFR-2, -3, c-KIT)	Renal Cell	placebo	**5.5** (* 2.8)	**19.3** (* 15.9)	[138]
			Hepatocellular	placebo	**5.5** (* 2.8)	**10.7** (* 7.9)	[139]
			Advanced thyroid	placebo	**10.8** (* 5.8)	-	[140,141]
Sunitinib (Sutent^®^)	2006	Tyrosine Kinase Inhibitor (PDGF-a, b, VEGFR-1, -2, -3, KIT, FLT-3, CSF-1R)	Pancreatic Neuo-endocrine	Placebo	**12.6** (* 5.8)	**38.6** (* 29.1)	[141]
			Gastrointestinal Stromal	Placebo	-	**18.5** (* 8.9)	[136]
Vandetanib	2011	Tyrosine Kinase Inhibitor (VEGFR-2, EGFR, RET)	Medullary Thyroid	Placebo	**30.5** (* 19.2)	-	[142]
Ziv-aflibercept	2012	Fusion protein (two human VEGF receptors connected by Fc domain)	Colorectal	FOLFIRI chemo (Folinic Acid, Fluorouracil, irinotecan)	**6.9** (* 4.7)	**13.5** (* 12.0)	[143]
Ramucirumab	2014	Human monoclonal antibody against VEGFR-2	Gastric	Paclitaxel	-	**9.6** (* 7.4)	[144,145]
			NSCLC	Docetaxel	**4.5** (* 3.0)	**10.5** (* 9.1)	[145]
			Colorectal	Placebo	**2.8** (* 1.6)	**8.5** (* 7.3)	[146]

* denotes overall survival (OS) or progression free survival (PFS) of the agents used alone whereas bolded numbers represent OS/PFS when used in combination with anti-angiogenic therapy.

## References

[1] Pereira R.D., de Long N.E., Wang R.C., Yazdi F.T., Holloway A.C., Raha S. (2015). Angiogenesis in the Placenta: The Role of Reactive Oxygen Species Signaling. BioMed Res. Int..

[2] Dangat K., Khaire A., Joshi S. (2020). Cross Talk of Vascular Endothelial Growth Factor and Neurotrophins in Mammary Gland Development. Growth Factors.

[3] Kumar P., Kumar S., Udupa E.P., Kumar U., Rao P., Honnegowda T. (2015). Role of Angiogenesis and Angiogenic Factors in Acute and Chronic Wound Healing. Plast. Aesthet. Res..

[4] Ucuzian A.A., Gassman A.A., East A.T., Greisler H.P. (2010). Molecular Mediators of Angiogenesis. J. Burn Care Res..

[5] Yokota Y., Nakajima H., Wakayama Y., Muto A., Kawakami K., Fukuhara S., Mochizuki N. (2015). Endothelial Ca^2+^ Oscillations Reflect VEGFR Signaling-Regulated Angiogenic Capacity in Vivo. eLife.

[6] Ghajar C.M., George S.C., Putnam A.J. (2008). Matrix Metalloproteinase Control of Capillary Morphogenesis. Crit. Rev. Eukaryot. Gene Expr..

[7] Sauteur L., Krudewig A., Herwig L., Ehrenfeuchter N., Lenard A., Affolter M., Belting H.G. (2014). Cdh5/VE-Cadherin Promotes Endothelial Cell Interface Elongation via Cortical Actin Polymerization during Angiogenic Sprouting. Cell Rep..

[8] Franco C.A., Jones M.L., Bernabeu M.O., Vion A.C., Barbacena P., Fan J., Mathivet T., Fonseca C.G., Ragab A., Yamaguchi T.P. (2016). Non-Canonical Wnt Signalling Modulates the Endothelial Shear Stress Flow Sensor in Vascular Remodelling. eLife.

[9] Chen Q., Jiang L., Li C., Hu D., Bu J.W., Cai D., Du J.L. (2012). Haemodynamics-Driven Developmental Pruning of Brain Vasculature in Zebrafish. PLoS Biol..

[10] Murakami M. (2012). Signaling Required for Blood Vessel Maintenance: Molecular Basis and Pathological Manifestations. Int. J. Vasc. Med..

[11] Bremnes R.M., Dønnem T., Al-Saad S., Al-Shibli K., Andersen S., Sirera R., Camps C., Marinez I., Busund L.T. (2011). The Role of Tumor Stroma in Cancer Progression and Prognosis: Emphasis on Carcinoma-Associated Fibroblasts and Non-Small Cell Lung Cancer. J. Thorac. Oncol..

[12] Murphy K.J., Chambers C.R., Herrmann D., Timpson P., Pereira B.A. (2021). Dynamic Stromal Alterations Influence Tumor-Stroma Crosstalk to Promote Pancreatic Cancer and Treatment Resistance. Cancers.

[13] Folkman J. (1971). Tumor Angiogenesis: Therapeutic Implications. N. Engl. J. Med..

[14] Hanahan D., Weinberg R.A. (2000). The Hallmarks of Cancer. Cell.

[15] Folkman J., Hanahan D. (1991). Switch to the Angiogenic Phenotype during Tumorigenesis. Princess Takamatsu Symp..

[16] Comerford K.M., Wallace T.J., Karhausen J., Louis N.A., Montalto M.C., Colgan S.P. (2002). Hypoxia-Inducible Factor-1-Dependent Regulation of the Multidrug Resistance (MDR1) Gene. Cancer Res..

[17] Wilkins S.E., Abboud M.I., Hancock R.L., Schofield C.J. (2016). Targeting Protein-Protein Interactions in the HIF System. ChemMedChem.

[18] Jeong J.-W., Bae M.-K., Ahn M.-Y., Kim S.-H., Sohn T.-K., Bae M.-H., Yoo M.-A., Song E.J., Lee K.-J., Kim K.-W. (2002). Regulation and Destabilization of HIF-1 by ARD1-Mediated Acetylation Quitin-Proteasome Pathway (Salceda and Caro The Association of PVHL and HIF-1 under nor-Moxic Conditions Is Triggered by the Posttranslational. Cell.

[19] Strowitzki M., Cummins E., Taylor C. (2019). Protein Hydroxylation by Hypoxia-Inducible Factor (HIF) Hydroxylases: Unique or Ubiquitous?. Cells.

[20] Yu A.Y., Frid M.G., Shimoda L.A., Wiener C.M., Stenmark K., Semenza G.L. (1998). Temporal, Spatial, and Oxygen-Regulated Expression of Hypoxia-Inducible Factor-1 in the Lung. Am. J. Physiol. Lung Cell. Mol. Physiol..

[21] McKeown S.R. (2014). Defining Normoxia, Physoxia and Hypoxia in Tumours—Implications for Treatment Response. Br. J. Radiol..

[22] Artemov A.V., Zhigalova N., Zhenilo S., Mazur A.M., Prokhortchouk E.B. (2018). VHL Inactivation without Hypoxia Is Sufficient to Achieve Genome Hypermethylation. Sci. Rep..

[23] Amelio I., Mancini M., Petrova V., Cairns R.A., Vikhreva P., Nicolai S., Marini A., Antonov A.A., le Quesne J., Baena Acevedo J.D. (2018). P53 Mutants Cooperate with HIF-1 in Transcriptional Regulation of Extracellular Matrix Components to Promote Tumor Progression. Proc. Natl. Acad. Sci. USA.

[24] Ravi R., Mookerjee B., Bhujwalla Z.M., Sutter C.H., Artemov D., Zeng Q., Dillehay L.E., Madan A., Semenza G.L., Bedi A. (2000). Regulation of Tumor Angiogenesis by P53-Induced Degradation of Hypoxia- Inducible Factor 1α. Genes Dev..

[25] Mohlin S., Hamidian A., von Stedingk K., Bridges E., Wigerup C., Bexell D., Påhlman S. (2015). PI3K-MTORC2 but Not PI3K-MTORC1 Regulates Transcription of HIF2A/EPAS1and Vascularization in Neuroblastoma. Cancer Res..

[26] Zhong H., Chiles K., Feldser D., Laughner E., Hanrahan C., Georgescu M.M., Simons J.W., Semenza G.L. (2000). Modulation of Hypoxia-Inducible Factor 1α Expression by the Epidermal Growth Factor/Phosphatidylinositol 3-Kinase/PTEN/AKT/FRAP Pathway in Human Prostate Cancer Cells: Implications for Tumor Angiogenesis and Therapeutics. Cancer Res..

[27] Lee B.I., Kim W.H., Jung J., Cho S.J., Park J.W., Kim J., Chung H.Y., Chang M.S., Nam S.Y. (2008). A Hypoxia-Independent up-Regulation of Hypoxia-Inducible Factor-1 by AKT Contributes to Angiogenesis in Human Gastric Cancer. Carcinogenesis.

[28] Stegeman H., Span P.N., Peeters W.J.M., Verheijen M.M.G., Grénman R., Meijer T.W.H., Kaanders J.H.A.M., Bussink J. (2016). Interaction between Hypoxia, AKT and HIF-1 Signaling in HNSCC and NSCLC: Implications for Future Treatment Strategies. Future Sci. OA.

[29] Ferrara N. (2004). Vascular Endothelial Growth Factor: Basic Science and Clinical Progress. Endocr. Rev..

[30] Claesson-Welsh L., Welsh M. (2013). VEGFA and Tumour Angiogenesis. J. Intern. Med..

[31] Li X., Eriksson U. (2001). Novel VEGF Family Members: VEGF-B, VEGF-C and VEGF-D. Int. J. Biochem. Cell Biol..

[32] Liang L., Yue Z., Du W., Li Y., Tao H., Wang D., Wang R., Huang Z., He N., Xie X. (2017). Molecular Imaging of Inducible VEGF Expression and Tumor Progression in a Breast Cancer Model. Cell. Physiol. Biochem..

[33] Chang J., Chaudhuri O. (2019). Beyond Proteases: Basement Membrane Mechanics and Cancer Invasion. J. Cell Biol..

[34] Zucker S., Vacirca J. (2004). Role of Matrix Metalloproteinases (MMPs) in Colorectal Cancer. Cancer Metastasis Rev..

[35] Hashizume H., Baluk P., Morikawa S., McLean J.W., Thurston G., Roberge S., Jain R.K., McDonald D.M. (2000). Openings between Defective Endothelial Cells Explain Tumor Vessel Leakiness. Am. J. Pathol..

[36] Hida K., Hida Y., Amin D.N., Flint A.F., Panigrahy D., Morton C.C., Klagsbrun M. (2004). Tumor-Associated Endothelial Cells with Cytogenetic Abnormalities. Cancer Res..

[37] Schaaf M.B., Garg A.D., Agostinis P. (2018). Defining the Role of the Tumor Vasculature in Antitumor Immunity and Immunotherapy Article. Cell Death Dis..

[38] Matsuda K., Ohga N., Hida Y., Muraki C., Tsuchiya K., Kurosu T., Akino T., Shih S.C., Totsuka Y., Klagsbrun M. (2010). Isolated Tumor Endothelial Cells Maintain Specific Character during Long-Term Culture. Biochem. Biophys. Res. Commun..

[39] Sievert W., Tapio S., Breuninger S., Gaipl U., Andratschke N., Trott K.R., Multhoff G. (2014). Adhesion Molecule Expression and Function of Primary Endothelial Cells in Benign and Malignant Tissues Correlates with Proliferation. PLoS ONE.

[40] Ohmura-Kakutani H., Akiyama K., Maishi N., Ohga N., Hida Y., Kawamoto T., Iida J., Shindoh M., Tsuchiya K., Shinohara N. (2014). Identification of Tumor Endothelial Cells with High Aldehyde Dehydrogenase Activity and a Highly Angiogenic Phenotype. PLoS ONE.

[41] Mehran R., Nilsson M., Khajavi M., Du Z., Cascone T., Wu H.K., Cortes A., Xu L., Zurita A., Schier R. (2014). Tumor Endothelial Markers Define Novel Subsets of Cancer-Specific Circulating Endothelial Cells Associated with Antitumor Efficacy. Cancer Res..

[42] Gatmaitan Z., Varticovski L., Ling L., Mikkelsen R., Steffan A.M., Arias I.M. (1996). Studies on Fenestral Contraction in Rat Liver Endothelial Cells in Culture. Am. J. Pathol..

[43] Levick J.R., Smaje L.H. (1987). An Analysis of the Permeability of a Fenestra. Microvasc. Res..

[44] Suarez S., Ballmer-Hofer K. (2001). VEGF Transiently Disrupts Gap Junctional Communication in Endothelial Cells. J. Cell Sci..

[45] Nimlamool W., Andrews R.M.K., Falk M.M. (2015). Connexin43 Phosphorylation by PKC and MAPK Signals VEGF-Mediated Gap Junction Internalization. Mol. Biol. Cell.

[46] Thuringer D. (2004). The Vascular Endothelial Growth Factor-Induced Disruption of Gap Junctions is Relayed by an Autocrine Communication via ATP Release in Coronary Capillary Endothelium. Ann. N. Y. Acad. Sci..

[47] Dvorak H.F., Senger D.R., Dvorak A.M. (1983). Fibrin as a Component of the Tumor Stroma: Origins and Biological Significance. Cancer Metastasis Rev..

[48] Xiang D., Feng Y., Wang J., Zhang X., Shen J., Zou R., Yuan Y. (2019). Platelet-derived Growth Factor-BB Promotes Proliferation and Migration of Retinal Microvascular Pericytes by Up-regulating the Expression of C-X-C Chemokine Receptor Types 4. Exp. Ther. Med..

[49] Lindahl P., Johansson B.R., Levéen P., Betsholtz C. (1997). Pericyte Loss and Microaneurysm Formation in PDGF-B-Deficient Mice. Science.

[50] Franco M., Roswall P., Cortez E., Hanahan D., Pietras K. (2011). Pericytes Promote Endothelial Cell Survival through Induction of Autocrine VEGF-Asignaling and Bcl-w Expression. Blood.

[51] Díaz-Flores L., Gutiérrez R., Madrid J.F., Varela H., Valladares F., Acosta E., Martín-Vasallo P., Díaz-Flores J. (2009). Pericytes. Morphofunction, Interactions and Pathology in a Quiescent and Activated Mesenchymal Cell Niche. Histol. Histopathol..

[52] Jain R.K. (1987). Transport of Molecules in the Tumor Interstitium: A Review. Cancer Res..

[53] Libutti S.K., Tamarkin L., Nilubol N. (2018). Targeting the Invincible Barrier for Drug Delivery in Solid Cancers: Interstitial Fluid Pressure. Oncotarget.

[54] Griffon-Etienne G., Boucher Y., Brekken C., Suit H.D., Jain R.K. (1999). Taxane-Induced Apoptosis Decompresses Blood Vessels and Lowers Interstitial Fluid Pressure in Solid Tumors: Clinical Implications. Cancer Res..

[55] Padera T.P., Stoll B.R., Tooredman J.B., Capen D., di Tomaso E., Jain R.K. (2004). Pathology: Cancer Cells Compress Intratumour Vessels 1 11672. Nature.

[56] Weis S.M., Cheresh D.A. (2005). Pathophysiological Consequences of VEGF-Induced Vascular Permeability. Nature.

[57] Nagy J.A., Dvorak A.M., Dvorak H.F. (2012). Vascular Hyperpermeability, Angiogenesis, and Stroma Generation. Cold Spring Harb. Perspect. Med..

[58] Stylianopoulos T., Martin J.D., Chauhan V.P., Jain S.R., Diop-Frimpong B., Bardeesy N., Smith B.L., Ferrone C.R., Hornicek F.J., Boucher Y. (2012). Causes, Consequences, and Remedies for Growth-Induced Solid Stress in Murine and Human Tumors. Proc. Natl. Acad. Sci. USA.

[59] Helmlinger G., Yuan F., Dellian M., Jain R.K. (1997). Interstitial PH and PO2 Gradients in Solid Tumors in Vivo: High-Resolution Measurements Reveal a Lack of Correlation. Nat. Med..

[60] Simonsen T.G., Lund K.V., Hompland T., Kristensen G.B., Rofstad E.K. (2018). DCE-MRI–Derived Measures of Tumor Hypoxia and Interstitial Fluid Pressure Predict Outcomes in Cervical Carcinoma. Int. J. Radiat. Oncol. Biol. Phys..

[61] Ferretti S., Allegrini P.R., Becquet M.M., McSheehy P.M.J. (2009). Tumor Interstitial Fluid Pressure as an Early-Response Marker for Anticancer Therapeutics. Neoplasia.

[62] Baish J.W., Netti P.A., Jain R.K. (1997). Transmural Coupling of Fluid Flow in Microcirculatory Network and Interstitium in Tumors. Microvasc. Res..

[63] Wu M., Frieboes H.B., Chaplain M.A.J., McDougall S.R., Cristini V., Lowengrub J.S. (2014). The Effect of Interstitial Pressure on Therapeutic Agent Transport: Coupling with the Tumor Blood and Lymphatic Vascular Systems. J. Theor. Biol..

[64] Jain R.K., Stylianopoulos T. (2010). Delivering Nanomedicine to Solid Tumors. Nat. Rev. Clin. Oncol..

[65] Bender L.H., Abbate F., Walters I.B. (2020). Intratumoral Administration of a Novel Cytotoxic Formulation with Strong Tissue Dispersive Properties Regresses Tumor Growth and Elicits Systemic Adaptive Immunity in in Vivo Models. Int. J. Mol. Sci..

[66] Sriraman S.K., Aryasomayajula B., Torchilin V.P. (2014). Barriers to Drug Delivery in Solid Tumors. Tissue Barriers.

[67] Fadaka A., Ajiboye B., Ojo O., Adewale O., Olayide I., Emuowhochere R. (2017). Biology of Glucose Metabolization in Cancer Cells. J. Oncol. Sci..

[68] Caswell D.R., Swanton C. (2017). The Role of Tumour Heterogeneity and Clonal Cooperativity in Metastasis, Immune Evasion and Clinical Outcome. BMC Med..

[69] Yang T., Wall E.M., Milne K., Theiss P., Watson P., Nelson B.H. (2007). CD8^+^ T Cells Induce Complete Regression of Advanced Ovarian Cancers by an Interleukin (IL)-2/IL-15–Dependent Mechanism. Clin. Cancer Res..

[70] Sonveaux P., Vegran F., Schroeder T., Wergin M.C., Verrax J., Rabbani Z.N., De Saedeleer C.J., Kennedy K.M., Diepart C., Jordan B.F. (2008). Targeting lactate-fueled respiration selectively kills hypoxic tumor cells in mice. J. Clin. Investig..

[71] Kennedy K.M., Scarbrough P.M., Ribeiro A., Richardson R., Yuan H., Sonveaux P., Landon C.D., Chi J.-T., Pizzo S., Schroeder T. (2013). Catabolism of Exogenous Lactate Reveals It as a Legitimate Metabolic Substrate in Breast Cancer. PLoS ONE.

[72] Wang Y., Bai C., Ruan Y., Liu M., Chu Q., Qiu L., Yang C., Li B. (2019). Coordinative metabolism of glutamine carbon and nitrogen in proliferating cancer cells under hypoxia. Nat. Commun..

[73] Pérez-Escuredo J., Dadhich R.K., Dhup S., Cacace A., Van Hée V., De Saedeleer C.J., Sboarina M., Rodriguez F., Fontenille M.-J., Brisson L. (2016). Lactate promotes glutamine uptake and metabolism in oxidative cancer cells. Cell Cycle.

[74] Gatenby R.A., Gawlinski E.T., Gmitro A.F., Kaylor B., Gillies R. (2006). Acid-Mediated Tumor Invasion: A Multidisciplinary Study. Cancer Res..

[75] Rauschner M., Lange L., Hüsing T., Reime S., Nolze A., Maschek M., Thews O., Riemann A. (2021). Impact of the acidic environment on gene expression and functional parameters of tumors in vitro and in vivo. J. Exp. Clin. Cancer Res..

[76] Riemann A., Schneider B., Gündel D., Stock C., Thews O., Gekle M. (2014). Acidic priming enhances metastatic potential of cancer cells. Pflügers Arch. Eur. J. Physiol..

[77] Wu H., Estrella V., Beatty M., Abrahams D., El-Kenawi A., Russell S., Ibrahim-Hashim A., Longo D.L., Reshetnyak Y.K., Moshnikova A. (2020). T-cells produce acidic niches in lymph nodes to suppress their own effector functions. Nat. Commun..

[78] Halcrow P.W., Geiger J.D., Chen X. (2021). Overcoming Chemoresistance: Altering pH of Cellular Compartments by Chloroquine and Hydroxychloroquine. Front. Cell Dev. Biol..

[79] Gray L.H., Conger A.D., Ebert M., Hornsey S., Scott O.C.A. (1953). The Concentration of Oxygen Dissolved in Tissues at the Time of Irradiation as a Factor in Radiotherapy. Br. J. Radiol..

[80] Price M., Heilbrun L., Kessel D. (2012). Effects of the oxygenation level on formation of different reactive oxygen species during photodynamic therapy. Photochem. Photobiol..

[81] Freitas I. (1988). Facing hypoxia: A must for photodynamic therapy. J. Photochem. Photobiol. B Biol..

[82] Nanayakkara A.K., Follit C.A., Chen G., Williams N.S., Vogel P.D., Wise J.G. (2018). Targeted inhibitors of P-glycoprotein increase chemotherapeutic-induced mortality of multidrug resistant tumor cells. Sci. Rep..

[83] Lv Y., Zhao S., Han J., Zheng L., Yang Z., Zhao L. (2015). Hypoxia-inducible factor-1α induces multidrug resistance protein in colon cancer. OncoTargets Ther..

[84] Vaidyanathan A., Sawers L., Gannon A.-L., Chakravarty P., Scott A.L., Bray S.E., Ferguson M.J., Smith G. (2016). ABCB1 (MDR1) induction defines a common resistance mechanism in paclitaxel- and olaparib-resistant ovarian cancer cells. Br. J. Cancer.

[85] Mirzaei S.A., Reiisi S., Tabari P.G., Shekari A., Aliakbari F., Azadfallah E., Elahian F. (2018). Broad blocking of MDR efflux pumps by acetylshikonin and acetoxyisovalerylshikonin to generate hypersensitive phenotype of malignant carcinoma cells. Sci. Rep..

[86] Samanta D., Gilkes D.M., Chaturvedi P., Xiang L., Semenza G.L. (2014). Hypoxia-inducible factors are required for chemotherapy resistance of breast cancer stem cells. Proc. Natl. Acad. Sci. USA.

[87] Chen J., Ding Z., Peng Y., Pan F., Li J., Zou L., Zhang Y., Liang H. (2014). HIF-1α Inhibition Reverses Multidrug Resistance in Colon Cancer Cells via Downregulation of MDR1/P-Glycoprotein. PLoS ONE.

[88] Thiery J.P., Acloque H., Huang R.Y.-J., Nieto M.A. (2009). Epithelial-Mesenchymal Transitions in Development and Disease. Cell.

[89] Saggar J.K., Tannock I.F. (2015). Chemotherapy Rescues Hypoxic Tumor Cells and Induces Their Reoxygenation and Repopulation—An Effect That Is Inhibited by the Hypoxia-Activated Prodrug TH-302. Clin. Cancer Res..

[90] Iyikesici M.S. (2020). Long-Term Survival Outcomes of Metabolically Supported Chemotherapy with Gemcitabine-Based or FOLFIRINOX Regimen Combined with Ketogenic Diet, Hyperthermia, and Hyperbaric Oxygen Therapy in Metastatic Pancreatic Cancer. Complement. Med. Res..

[91] Alagoz T., Buller R.E., Anderson B., Terrell K.L., Squatrito R.C., Niemann T.H., Tatman D.J., Jebson P. (1995). Evaluation of hyperbaric oxygen as a chemosensitizer in the treatment of epithelial ovarian cancer in xenografts in mice. Cancer.

[92] Takiguchi N., Saito N., Nunomura M., Kouda K., Oda K., Furuyama N., Nakajima N. (2000). Use of 5-FU plus hyperbaric oxygen for treating malignant tumors: Evaluation of antitumor effect and measurement of 5-FU in individual organs. Cancer Chemother. Pharmacol..

[93] Khouzam R.A., Brodaczewska K., Filipiak A., Zeinelabdin N.A., Buart S., Szczylik C., Kieda C., Chouaib S. (2021). Tumor Hypoxia Regulates Immune Escape/Invasion: Influence on Angiogenesis and Potential Impact of Hypoxic Biomarkers on Cancer Therapies. Front. Immunol..

[94] Clambey E.T., McNamee E.N., Westrich J.A., Glover L.E., Campbell E.L., Jedlicka P., de Zoeten E.F., Cambier J.C., Stenmark K.R., Colgan S.P. (2012). Hypoxia-Inducible Factor-1 Alpha-Dependent Induction of FoxP3 Drives Regulatory T-Cell Abundance and Function during Inflammatory Hypoxia of the Mucosa. Proc. Natl. Acad. Sci. USA.

[95] Barsoum I.B., Smallwood C.A., Siemens D.R., Graham C.H. (2014). A Mechanism of Hypoxia-Mediated Escape from Adaptive Immunity in Cancer Cells. Cancer Res..

[96] Noman M.Z., Buart S., van Pelt J., Richon C., Hasmim M., Leleu N., Suchorska W.M., Jalil A., Lecluse Y., el Hage F. (2009). The Cooperative Induction of Hypoxia-Inducible Factor-1 Alpha and STAT3 during Hypoxia Induced an Impairment of Tumor Susceptibility to CTL-Mediated Cell Lysis. J. Immunol. (Baltim. Md. 1950).

[97] Damgaci S., Ibrahim-Hashim A., Enriquez-Navas P.M., Pilon-Thomas S., Guvenis A., Gillies R.J. (2018). Hypoxia and Acidosis: Immune Suppressors and Therapeutic Targets. Immunology.

[98] Choukèr A., Thiel M., Lukashev D., Ward J.M., Kaufmann I., Apasov S., Sitkovsky M.V., Ohta A. (2008). Critical Role of Hypoxia and A2A Adenosine Receptors in Liver Tissue-Protecting Physiological Anti-Inflammatory Pathway. Mol. Med..

[99] Mpekris F., Voutouri C., Baish J.W., Duda D.G., Munn L.L., Stylianopoulos T., Jain R.K. (2020). Combining Microenvironment Normalization Strategies to Improve Cancer Immunotherapy. Proc. Natl. Acad. Sci. USA.

[100] Chouaib S., Noman M.Z., Kosmatopoulos K., Curran M.A. (2017). Hypoxic Stress: Obstacles and Opportunities for Innovative Immunotherapy of Cancer. Oncogene.

[101] Torres N., Regge M.V., Secchiari F., Friedrich A.D., Spallanzani R.G., Raffo Iraolagoitia X.L., Núñez S.Y., Sierra J.M., Ziblat A., Santilli M.C. (2020). Restoration of Antitumor Immunity through Anti-MICA Antibodies Elicited with a Chimeric Protein. J. ImmunoTherapy Cancer.

[102] Labiano S., Palazon A., Melero I. (2015). Immune Response Regulation in the Tumor Microenvironment by Hypoxia. Semin. Oncol..

[103] Katsuno Y., Lamouille S., Derynck R. (2013). TGF-β Signaling and Epithelial-Mesenchymal Transition in Cancer Progression. Curr. Opin. Oncol..

[104] Borriello L., Nakata R., Sheard M.A., Fernandez G.E., Sposto R., Malvar J., Blavier L., Shimada H., Asgharzadeh S., Seeger R.C. (2017). Cancer-Associated Fibroblasts Share Characteristics and Protumorigenic Activity with Mesenchymal Stromal Cells. Cancer Res..

[105] Tran C.W., Gold M.J., Garcia-Batres C., Tai K., Elford A.R., Himmel M.E., Elia A.J., Ohashi P.S. (2020). Hypoxia-Inducible Factor 1 Alpha Limits Dendritic Cell Stimulation of CD8 T Cell Immunity. PLoS ONE.

[106] Weigert A., Weichand B., Sekar D., Sha W., Hahn C., Mora J., Ley S., Essler S., Dehne N., Brüne B. (2012). HIF-1α Is a Negative Regulator of Plasmacytoid DC Development in Vitro and in Vivo. Blood.

[107] Colliez F., Gallez B., Jordan B.F. (2017). Assessing Tumor Oxygenation for Predicting Outcome in Radiation Oncology: A Review of Studies Correlating Tumor Hypoxic Status and Outcome in the Preclinical and Clinical Settings. Front. Oncol..

[108] Yeh J.J., Kim W.Y. (2015). Targeting Tumor Hypoxia with Hypoxia-Activated Prodrugs. J. Clin. Oncol..

[109] Thews O., Vaupel P. (2015). Spatial Oxygenation Profiles in Tumors during Normo- and Hyperbaric Hyperoxia. Strahlenther. Und Onkol..

[110] Gainer J.L. (2012). Increasing Oxygen in Hypoxic Tumors. Clin. Exp. Pharmacol..

[111] Eisenbrey J.R., Shraim R., Liu J.B., Li J., Stanczak M., Oeffinger B., Leeper D.B., Keith S.W., Jablonowski L.J., Forsberg F. (2018). Sensitization of Hypoxic Tumors to Radiation Therapy Using Ultrasound-Sensitive Oxygen Microbubbles. Int. J. Radiat. Oncol. Biol. Phys..

[112] Cheng Y., Cheng H., Jiang C., Qiu X., Wang K., Huan W., Yuan A., Wu J., Hu Y. (2015). Perfluorocarbon Nanoparticles Enhance Reactive Oxygen Levels and Tumour Growth Inhibition in Photodynamic Therapy. Nat. Commun..

[113] Kim K.J., Li B., Winer J., Armanini M., Gillett N., Phillips H.S., Ferrara N. (1993). Inhibition of Vascular Endothelial Growth Factor-Induced Angiogenesis Suppresses Tumour Growth in Vivo. Nature.

[114] Van Beijnum J.R., Nowak-Sliwinska P., Huijbers E.J.M., Thijssen V.L., Griffioen A.W. (2015). The Great Escape; the Hallmarks of Resistance to Antiangiogenic Therapy. Pharmacol. Rev..

[115] Jain R.K. (2014). Antiangiogenesis Strategies Revisited: From Starving Tumors to Alleviating Hypoxia. Cancer Cell.

[116] Eales K.L., Hollinshead K.E.R., Tennant D.A. (2016). Hypoxia and Metabolic Adaptation of Cancer Cells. Oncogenesis.

[117] Godet I., Shin Y.J., Ju J.A., Ye I.C., Wang G., Gilkes D.M. (2019). Fate-Mapping Post-Hypoxic Tumor Cells Reveals a ROS-Resistant Phenotype That Promotes Metastasis. Nat. Commun..

[118] Verduzco D., Lloyd M., Xu L., Ibrahim-Hashim A., Balagurunathan Y., Gatenby R.A., Gillies R.J. (2015). Intermittent Hypoxia Selects for Genotypes and Phenotypes That Increase Survival, Invasion, and Therapy Resistance. PLoS ONE.

[119] Jain R.K. (2005). Normalization of Tumor Vasculature: An Emerging Concept in Antiangiogenic Therapy. Science.

[120] Dor Y., Porat R., Keshet E. (2001). Vascular Endothelial Growth Factor and Vascular Adjustments to Perturbations in Oxygen Homeostasis. Am. J. Physiol. Cell Physiol..

[121] Martin J.D., Seano G., Jain R.K. (2019). Normalizing Function of Tumor Vessels: Progress, Opportunities, and Challenges. Annu. Rev. Physiol..

[122] Tewari K.S., Sill M.W., Long H.J., Penson R.T., Huang H., Ramondetta L.M., Landrum L.M., Oaknin A., Reid T.J., Leitao M.M. (2014). Improved Survival with Bevacizumab in Advanced Cervical Cancer. N. Engl. J. Med..

[123] Kabbinavar F.F., Hambleton J., Mass R.D., Hurwitz H.I., Bergsland E., Sarkar S. (2005). Combined Analysis of Efficacy: The Addition of Bevacizumab to Fluorouracil/Leucovorin Improves Survival for Patients with Metastatic Colorectal Cancer. J. Clin. Oncol..

[124] Sandler A., Gray R., Perry M.C., Brahmer J., Schiller J.H., Dowlati A., Lilenbaum R., Johnson D.H. (2006). Paclitaxel-Carboplatin Alone or with Bevacizumab for Non-Small-Cell Lung Cancer. N. Engl. J. Med..

[125] Perren T.J., Swart A.M., Pfisterer J., Ledermann J.A., Pujade-Lauraine E., Kristensen G., Carey M.S., Beale P., Cervantes A., Kurzeder C. (2011). A Phase 3 Trial of Bevacizumab in Ovarian Cancer. N. Engl. J. Med..

[126] Escudier B., Pluzanska A., Koralewski P., Ravaud A., Bracarda S., Szczylik C., Chevreau C., Filipek M., Melichar B., Bajetta E. (2007). Bevacizumab plus Interferon Alfa-2a for Treatment of Metastatic Renal Cell Carcinoma: A Randomised, Double-Blind Phase III Trial. Lancet.

[127] Escudier B., Bellmunt J., Négrier S., Bajetta E., Melichar B., Bracarda S., Ravaud A., Golding S., Jethwa S., Sneller V. (2010). Phase III Trial of Bevacizumab plus Interferon Alfa-2a in Patients with Metastatic Renal Cell Carcinoma (AVOREN): Final Analysis of Overall Survival. J. Clin. Oncol..

[128] Rini B.I., Plimack E.R., Stus V., Gafanov R., Hawkins R., Nosov D., Pouliot F., Alekseev B., Soulières D., Melichar B. (2019). Pembrolizumab plus Axitinib versus Sunitinib for Advanced Renal-Cell Carcinoma. N. Engl. J. Med..

[129] Abou-Alfa G.K., Meyer T., Cheng A.L., El-Khoueiry A.B., Rimassa L., Ryoo B.Y., Cicin I., Merle P., Chen Y.H., Park J.W. (2018). Cabozantinib in Patients with Advanced and Progressing Hepatocellular Carcinoma. N. Engl. J. Med..

[130] Elisei R., Schlumberger M.J., Müller S.P., Schöffski P., Brose M.S., Shah M.H., Licitra L., Jarzab B., Medvedev V., Kreissl M.C. (2013). Cabozantinib in Progressive Medullary Thyroid Cancer. J. Clin. Oncol..

[131] Beaver J., Park B.H. (2013). The BOLERO-2 Trial: The Addition of Everolimus to Exemestane in the Treatment of Postmenopausal Hormone Receptor-Positive Advanced Breast Cancer. Future Oncol..

[132] Motzer R.J., Hutson T.E., Glen H., Michaelson M.D., Molina A., Eisen T., Jassem J., Zolnierek J., Maroto J.P., Mellado B. (2015). Lenvatinib, Everolimus, and the Combination in Patients with Metastatic Renal Cell Carcinoma: A Randomised, Phase 2, Open-Label, Multicentre Trial. Lancet Oncol..

[133] Sternberg C.N., Davis I.D., Mardiak J., Szczylik C., Lee E., Wagstaff J., Barrios C.H., Salman P., Gladkov O.A., Kavina A. (2010). Pazopanib in Locally Advanced or Metastatic Renal Cell Carcinoma: Results of a Randomized Phase III Trial. J. Clin. Oncol..

[134] van der Graaf W.T.A., Blay J.Y., Chawla S.P., Kim D.W., Bui-Nguyen B., Casali P.G., Schöffski P., Aglietta M., Staddon A.P., Beppu Y. (2012). Pazopanib for Metastatic Soft-Tissue Sarcoma (PALETTE): A Randomised, Double-Blind, Placebo-Controlled Phase 3 Trial. Lancet.

[135] Grothey A., van Cutsem E., Sobrero A., Siena S., Falcone A., Ychou M., Humblet Y., Bouché O., Mineur L., Barone C. (2013). Regorafenib Monotherapy for Previously Treated Metastatic Colorectal Cancer (CORRECT): An International, Multicentre, Randomised, Placebo-Controlled, Phase 3 Trial. Lancet.

[136] Demetri G.D., Reichardt P., Kang Y.K., Blay J.Y., Rutkowski P., Gelderblom H., Hohenberger P., Leahy M., von Mehren M., Joensuu H. (2013). Effi Cacy and Safety of Regorafenib for Advanced Gastrointestinal Stromal Tumours after Failure of Imatinib and Sunitinib (GRID): An International, Multicentre, Randomised, Placebo-Controlled, Phase 3 Trial. Lancet.

[137] Bruix J., Qin S., Merle P., Granito A., Huang Y.H., Bodoky G., Pracht M., Yokosuka O., Rosmorduc O., Breder V. (2017). Regorafenib for Patients with Hepatocellular Carcinoma Who Progressed on Sorafenib Treatment (RESORCE): A Randomised, Double-Blind, Placebo-Controlled, Phase 3 Trial. Lancet.

[138] Escudier B., Eisen T., Stadler W.M., Szczylik C., Oudard S., Siebels M., Negrier S., Chevreau C., Solska E., Desai A.A. (2007). Sorafenib in Advanced Clear-Cell Renal-Cell Carcinoma. N. Engl. J. Med..

[139] Llovet J.M., Ricci S., Mazzaferro V., Hilgard P., Gane E., Blanc J.-F., Cosme de Oliveira A., Santoro A., Raoul J.-L., Forner A. (2008). Sorafenib in Advanced Hepatocellular Carcinoma. N. Engl. J. Med..

[140] Brose M.S., Nutting C.M., Jarzab B., Elisei R., Siena S., Bastholt L., de La Fouchardiere C., Pacini F., Paschke R., Shong Y.K. (2014). Sorafenib in Radioactive Iodine-Refractory, Locally Advanced or Metastatic Diff Erentiated Thyroid Cancer: A Randomised, Double-Blind, Phase 3 Trial. Lancet.

[141] Faivre S., Niccoli P., Castellano D., Valle J.W., Hammel P., Raoul J.L., Vinik A., van Cutsem E., Bang Y.J., Lee S.H. (2017). Sunitinib in Pancreatic Neuroendocrine Tumors: Updated Progression-Free Survival and Final Overall Survival from a Phase III Randomized Study. Ann. Oncol..

[142] Wells S.A., Robinson B.G., Gagel R.F., Dralle H., Fagin J.A., Santoro M., Baudin E., Elisei R., Jarzab B., Vasselli J.R. (2012). Vandetanib in Patients with Locally Advanced or Metastatic Medullary Thyroid Cancer: A Randomized, Double-Blind Phase III Trial. J. Clin. Oncol..

[143] van Cutsem E., Tabernero J., Lakomy R., Prenen H., Prausová J., Macarulla T., Ruff P., van Hazel G.A., Moiseyenko V., Ferry D. (2012). Addition of Aflibercept to Fluorouracil, Leucovorin, and Irinotecan Improves Survival in a Phase III Randomized Trial in Patients with Metastatic Colorectal Cancer Previously Treated with an Oxaliplatin-Based Regimen. J. Clin. Oncol..

[144] Wilke H., Muro K., van Cutsem E., Oh S.C., Bodoky G., Shimada Y., Hironaka S., Sugimoto N., Lipatov O., Kim T.Y. (2014). Ramucirumab plus Paclitaxel versus Placebo plus Paclitaxel in Patients with Previously Treated Advanced Gastric or Gastro-Oesophageal Junction Adenocarcinoma (RAINBOW): A Double-Blind, Randomised Phase 3 Trial. Lancet Oncol..

[145] Garon E.B., Ciuleanu T.E., Arrieta O., Prabhash K., Syrigos K.N., Goksel T., Park K., Gorbunova V., Kowalyszyn R.D., Pikiel J. (2014). Ramucirumab plus Docetaxel versus Placebo plus Docetaxel for Second-Line Treatment of Stage IV Non-Small-Cell Lung Cancer after Disease Progression on Platinum-Based Therapy (REVEL): A Multicentre, Double-Blind, Randomised Phase 3 Trial. Lancet.

[146] Zhu A.X., Kang Y.K., Yen C.J., Finn R.S., Galle P.R., Llovet J.M., Assenat E., Brandi G., Pracht M., Lim H.Y. (2019). Ramucirumab after Sorafenib in Patients with Advanced Hepatocellular Carcinoma and Increased α-Fetoprotein Concentrations (REACH-2): A Randomised, Double-Blind, Placebo-Controlled, Phase 3 Trial. Lancet Oncol..

[147] Bais C., Mueller B., Brady M.F., Mannel R.S., Burger R.A., Wei W., Marien K.M., Kockx M.M., Husain A., Birrer M.J. (2017). Tumor Microvessel Density as a Potential Predictive Marker for Bevacizumab Benefit: GOG-0218 Biomarker Analyses. J. Natl. Cancer Inst..

[148] Martin J.D., Fukumura D., Duda D.G., Boucher Y., Jain R.K. (2016). Reengineering the Tumor Microenvironment to Alleviate Hypoxia and Overcome Cancer Heterogeneity. Cold Spring Harb. Perspect. Med..

[149] Tolaney S.M., Boucher Y., Duda D.G., Martin J.D., Seano G., Ancukiewicz M., Barry W.T., Goel S., Lahdenrata J., Isakoff S.J. (2015). Role of Vascular Density and Normalization in Response to Neoadjuvant Bevacizumab and Chemotherapy in Breast Cancer Patients. Proc. Natl. Acad. Sci. USA.

[150] Jayson G.C., Kerbel R., Ellis L.M., Harris A.L. (2016). Antiangiogenic Therapy in Oncology: Current Status and Future Directions. Lancet.

[151] Lawler J. (1986). The Structural and Functional Properties of Thrombospondin. Blood.

[152] Jiménez B., Volpert O.V., Crawford S.E., Febbraio M., Silverstein R.L., Bouck N. (2000). Signals Leading to Apoptosis-Dependent Inhibition of Neovascularization by Thrombospondin-1. Nat. Med..

[153] Bocci G., Francia G., Man S., Lawler J., Kerbel R.S. (2003). Thrombospondin 1, a Mediator of the Antiangiogenic Effects of Low-Dose Metronomic Chemotherapy. Proc. Natl. Acad. Sci. USA.

[154] Goel S., Gupta N., Walcott B.P., Snuderl M., Kesler C.T., Kirkpatrick N.D., Heishi T., Huang Y., Martin J.D., Ager E. (2013). Effects of Vascular-Endothelial Protein Tyrosine Phosphatase Inhibition on Breast Cancer Vasculature and Metastatic Progression. J. Natl. Cancer Inst..

[155] Peterson T.E., Kirkpatrick N.D., Huang Y., Farrar C.T., Marijt K.A., Kloepper J., Datta M., Amoozgar Z., Seano G., Jung K. (2016). Dual Inhibition of Ang-2 and VEGF Receptors Normalizes Tumor Vasculature and Prolongs Survival in Glioblastoma by Altering Macrophages. Proc. Natl. Acad. Sci. USA.

[156] Tian L., Goldstein A., Wang H., Lo H.C., Kim I.S., Welte T., Sheng K., Dobrolecki L.E., Zhang X., Putluri N. (2017). Mutual Regulation of Tumour Vessel Normalization and Immunostimulatory Reprogramming. Nature.

[157] Kłosowska-Wardȩga A., Hasumi Y., Burmakin M., Åhgren A., Stuhr L., Moen I., Reed R.K., Rubin K., Hellberg C., Heldin C.H. (2009). Combined Anti-Angiogenic Therapy Targeting PDGF and Vegf Receptors Lowers the Interstitial Fluid Pressure in a Murine Experimental Carcinoma. PLoS ONE.

[158] Matuszewska K., Santry L.A., van Vloten J.P., AuYeung A.W.K., Major P.P., Lawler J., Wootton S.K., Bridle B.W., Petrik J. (2019). Combining Vascular Normalization with an Oncolytic Virus Enhances Immunotherapy in a Preclinical Model of Advanced-Stage Ovarian Cancer. Clin. Cancer Res..

[159] Shrimali R.K., Yu Z., Theoret M.R., Chinnasamy D., Restifo N.P., Rosenberg S.A. (2010). Antiangiogenic Agents Can Increase Lymphocyte Infiltration into Tumor and Enhance the Effectiveness of Adoptive Immunotherapy of Cancer. Cancer Res..

[160] Goel S., Wong A.H.K., Jain R.K. (2012). Vascular Normalization as a Therapeutic Strategy for Malignant and Nonmalignant Disease. Cold Spring Harb. Perspect. Med..

